# Supplementary material for: Three-degrees-of-freedom orientation manipulation of small untethered robots with a single anisotropic soft magnet
Source: Nat Commun. 2023 Nov 18;14:7491. doi: 10.1038/s41467-023-42783-5 (PMC10657469; doi:10.1038/s41467-023-42783-5)
Supplement: Supplementary file 1 — Supplementary Information [file 41467_2023_42783_MOESM1_ESM.pdf]

Supplementary Information

Three-degrees-of-freedom Orientation Manipulation of Small Untethered Robots with an On-board Anisotropic Soft Magnet

Heng Wang[1,2*], Junhao Cui[1,2], Kuan Tian[2], Yuxiang Han[2]

[1] The authors contributed equally to this work.

[2] Shien-Ming Wu School of Intelligent Engineering, South China University of Technology, Guangzhou, Guangdong, China.

* To whom correspondence should be addressed. Email: wanghengscut@scut.edu.cn

Supplementary Figures

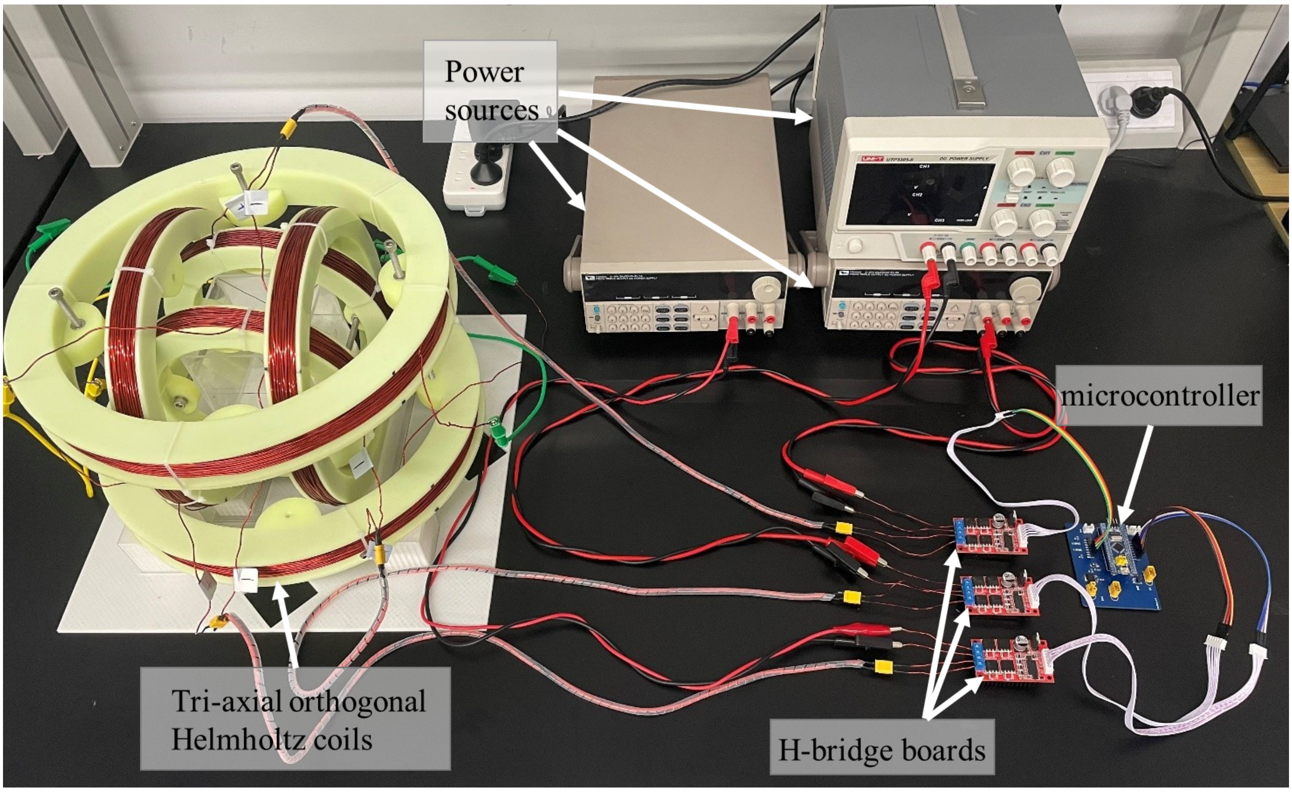

Supplementary Fig. 1 | Experimental setup. The magnetic actuation setup is built with tri-axial Helmholtz coils in an orthogonal configuration. The radius of inner coil, middle coil and outer coil are 90 mm, 120 mm and 160 mm, respectively. The capability of magnetic field generation at the center of the workspace is 22.43×10^{-5} T/A, 16.96×10^{-5} T/A and 22.96×10^{-5} T/A for the inner coil, middle coil and outer coil, respectively. The current of the Helmholtz coil is supplied by three DC power sources (rated voltage: 30V, rated current: 3A).

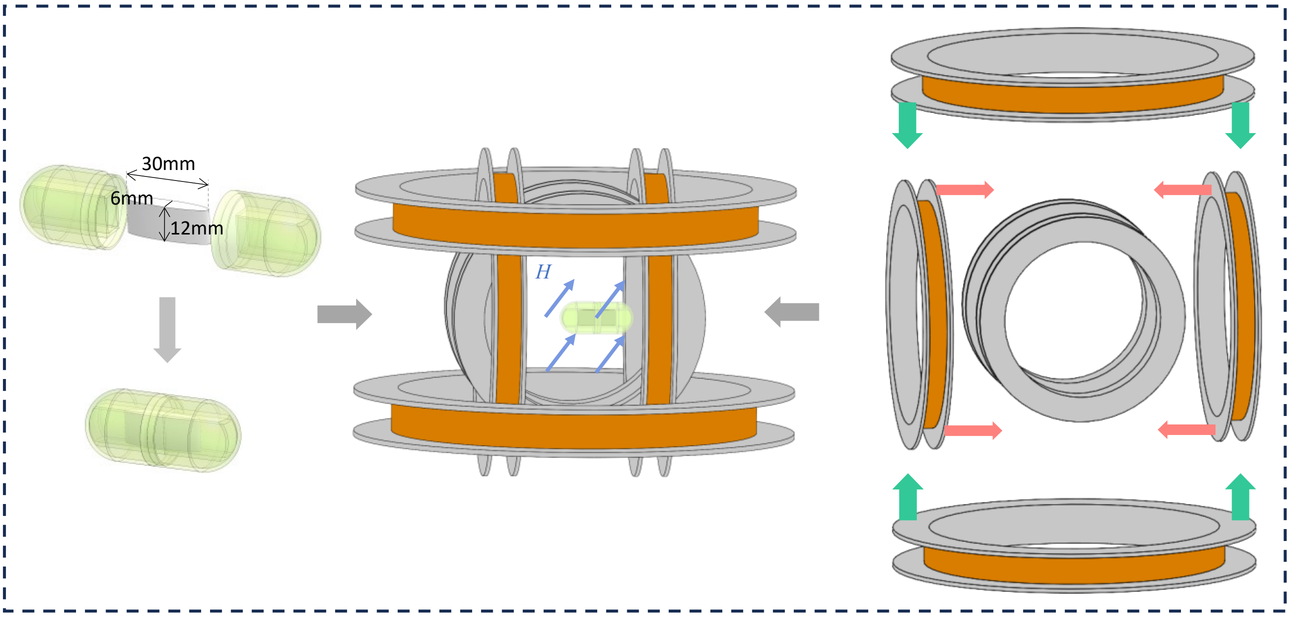

Supplementary Fig. 2 | Orthogonal Helmholtz coils. Three orthogonally located Helmholtz coils can generate a uniform magnetic field in any direction in the middle workspace. This experimental setup is used to validate the theoretical model of magnetic torques on an anisotropic soft magnet, to investigate the orientation stability of the soft magnet, and to demonstrate 3-DoF orientation control of the soft-magnet robot.

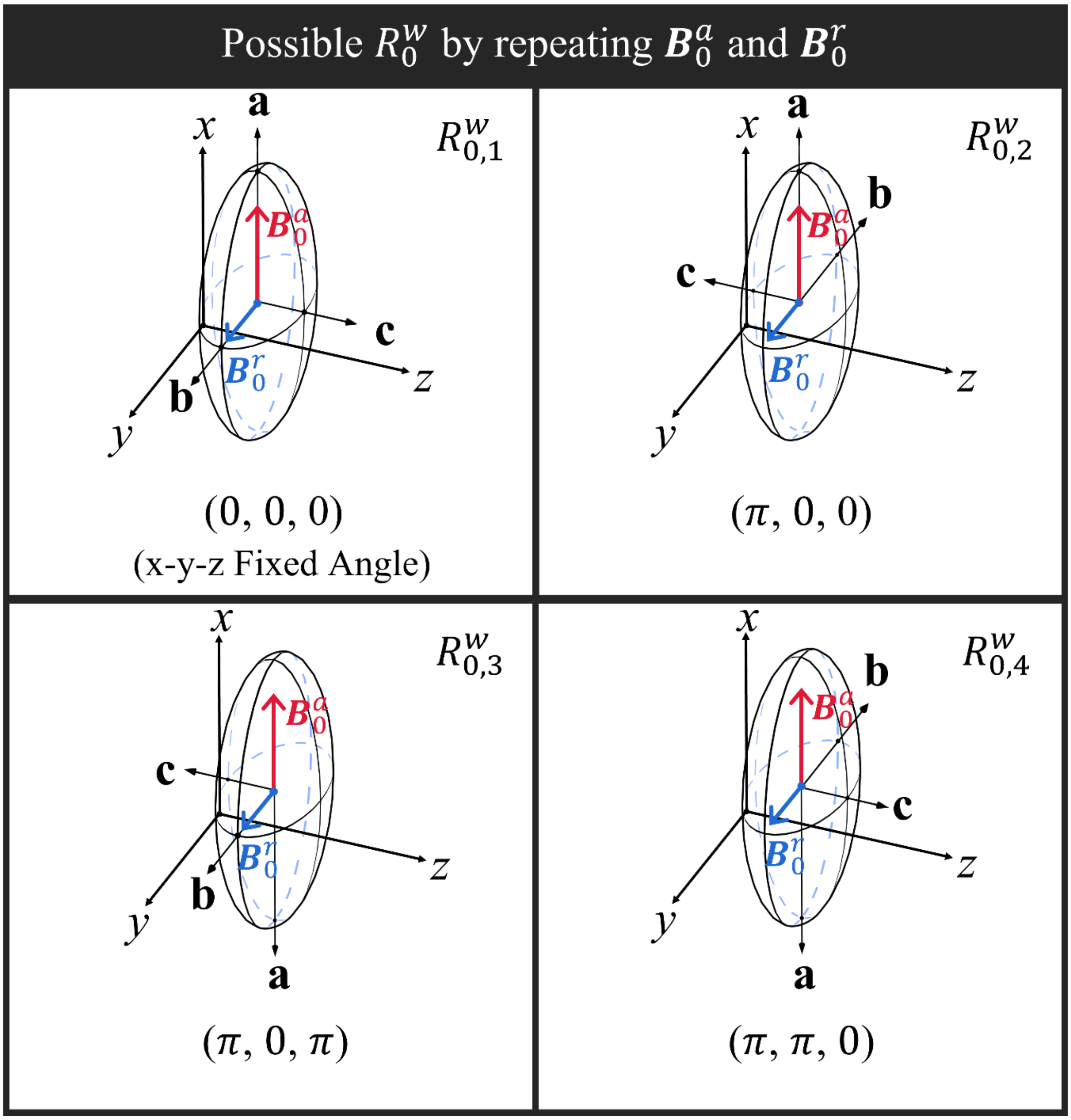

Supplementary Fig. 3 | Ambiguity of initial orientation. In 3-DoF orientation control, magnetic fields $\mathbf{B}_0^a$ and $\mathbf{B}_0^r$ are alternatingly applied at a frequency of 10 Hz and a duty cycle of 50% to set the initial orientation. For example, the initial orientation is set as the same as the world frame (i.e. $R_0^w = I$). To make the soft magnet at an unknown orientation reach the initial orientation, $\mathbf{B}_0^a$ field is applied along **x**-axis of the world frame while $\mathbf{B}_0^r$ field is applied along **y**-axis of the world frame. As a result, the **a**-axis of the soft magnet aligns with $\mathbf{B}_0^a$ (global **x**-axis) and the b-axis of the soft magnet aligns with $\mathbf{B}_0^r$ (global **y**-axis). However, whether the positive or negative direction of **a**- and **b**-axis of the soft magnet is aligned with the desired direction depends on the initial angle between the applied field and **a**- or **b**-axis (Problem 2 described in the section of "Alternating and Open-loop Control of 3-DoF Orientation of the Soft-magnet Robot" in the main text). Therefore, there are four possible orientations after the initialization with $\mathbf{B}_0^a$ and $\mathbf{B}_0^r$, i.e. $R_{0,1}^w$, $R_{0,2}^w$, $R_{0,3}^w$, $R_{0,4}^w$, as shown in the figure.

After completing the initialization, we need external observation to determine which of the four possible orientations the soft magnet is actually at. This determination only needs rough identification from four possible orientation choices rather than accurate and numerical orientation feedback, making the proposed control method still an open-loop approach.

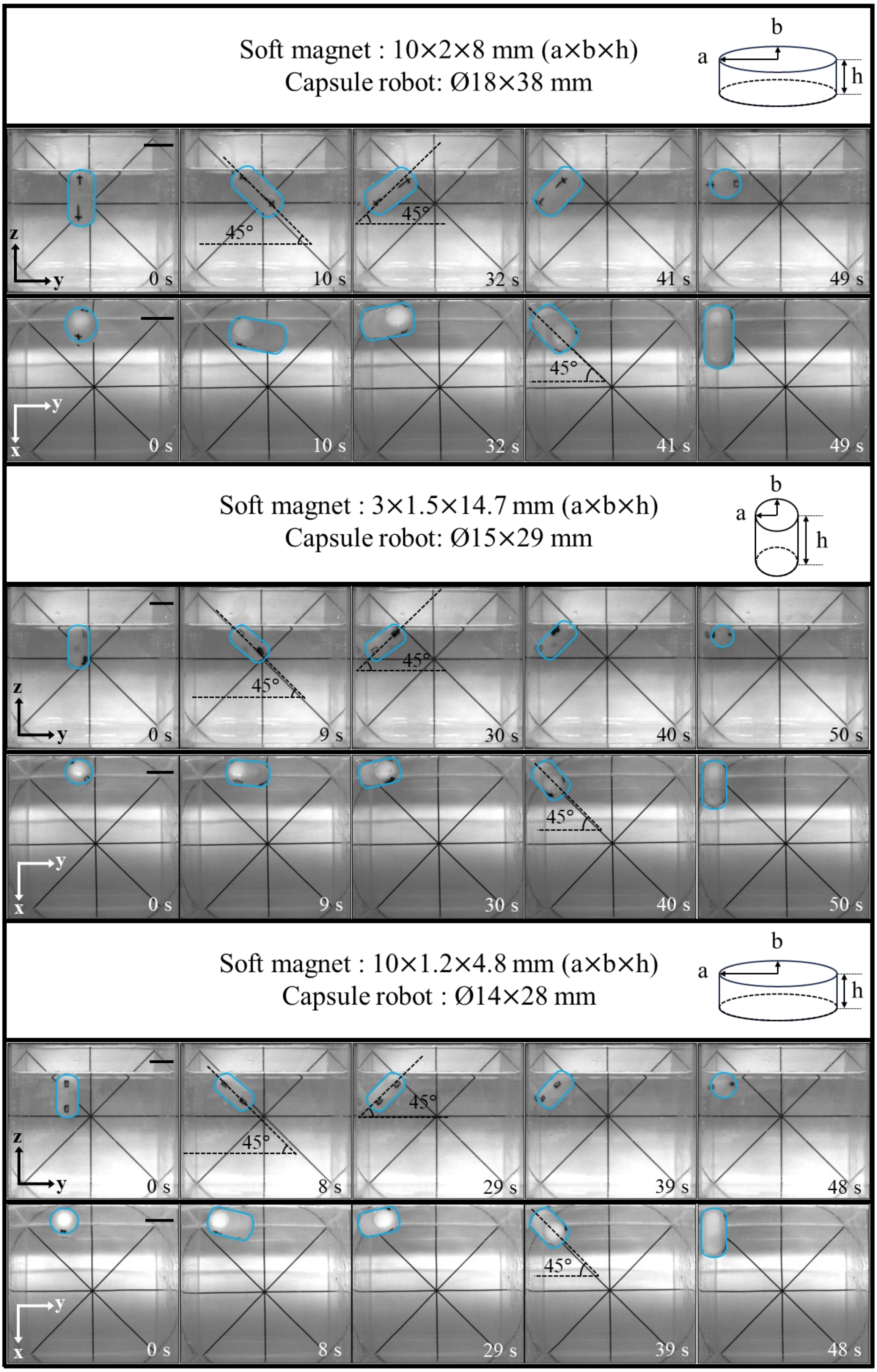

Soft magnet : 10×2×8 mm (a×b×h)
Capsule robot: Ø18×38 mm
a
b
h
45°
z
y
x
0 s
10 s
32 s
41 s
49 s
Soft magnet : 3×1.5×14.7 mm (a×b×h)
Capsule robot: Ø15×29 mm
9 s
30 s
40 s
50 s
Soft magnet : 10×1.2×4.8 mm (a×b×h)
Capsule robot : Ø14×28 mm
8 s
29 s
39 s
48 s

Supplementary Fig. 4 | 3-DoF orientation manipulation of soft-magnet robots of different sizes. Experiments of 3-DoF orientation manipulation are conducted with smaller capsule robots down to the size of commercial capsule endoscopes ($\emptyset 14$ mm $\times$ 28 mm) and smaller soft magnets down to the size of $10 \times 1.2 \times 4.8$ mm. The orientation path is the same as that in Fig. 6 (b) in the paper. It is shown that the proposed soft-magnet actuation method can achieve accurate 3-DoF orientation manipulation of small capsule robots. The scale bars indicate 2 cm.

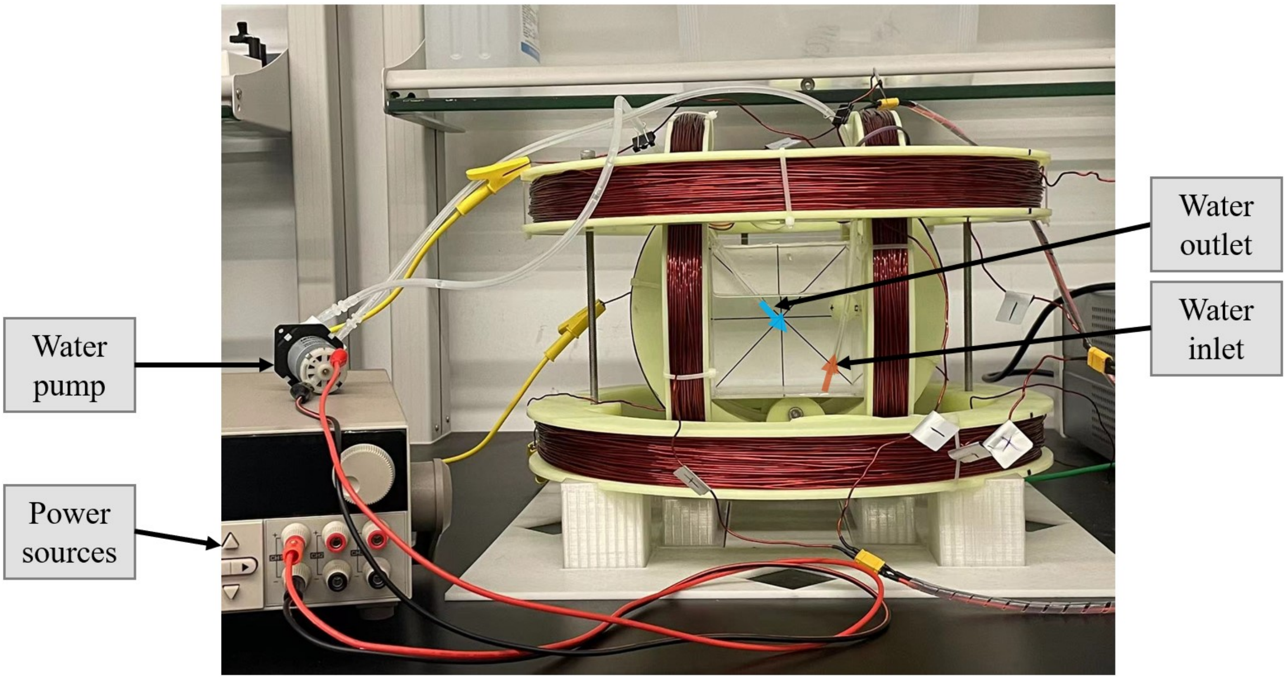

Supplementary Fig. 5 | Water circulation system to introduce flow disturbance. The water in the tank to suspend the capsule robot is extracted by a water pump and meanwhile pumped back to the tank. The water inlet (red arrow) is at the bottom of the tank and the water outlet (blue arrow) is near the surface. The water circulation causes a flow disturbance to the orientation manipulation of the capsule robot. The water flow rate is set to be 100mL/min and the volume of water in the tank is about 750mL.

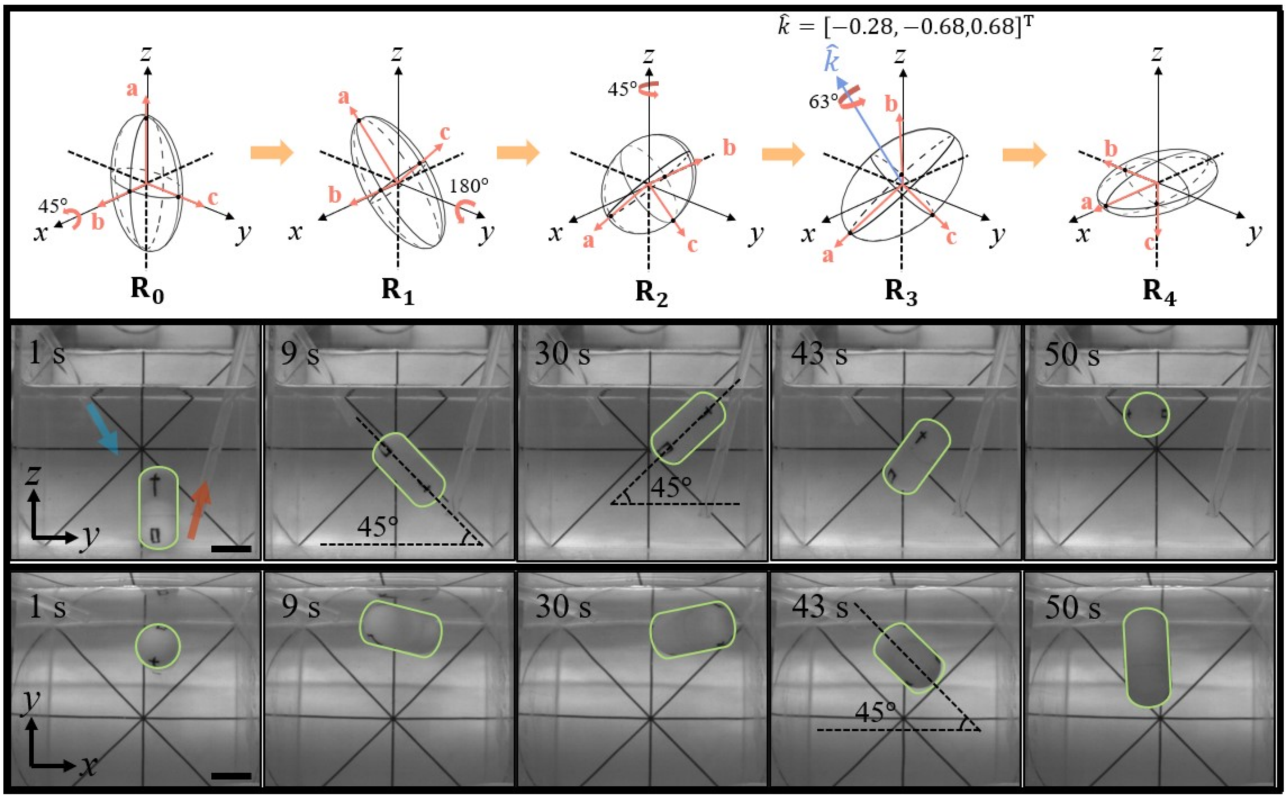

Supplementary Fig. 6 | 3-DoF orientation manipulation in the environmental flow field. The experiment of 3-DoF orientation manipulation in the environmental flow field is carried out using a water circulation system (Supplementary Fig. 5). The orientation path is the same as that in Fig. 6 (b) in the paper. The same control accuracy is achieved as the case without flow disturbance, which shows the robustness of the proposed soft-magnet manipulation method. The scale bars indicate 2 cm.

Supplementary Tables

Rotation about x-axis for 45°							
Current (A)	Total video Frames	Total video duration (s)	Frame rate (s^{-1})	Effective rotating frames	Rotating time (s)	Theoretical maximum torque ($\times10^{-5}$ N•m)	Experimental maximum torque ($\times10^{-5}$ N•m)
0.1	902	15.2	59.34	750	12.64	0.005	0.001
0.2	342	5.7	60.00	285	4.75	0.017	0.010
0.3	204	3.4	60.00	182	3.03	0.039	0.025
0.4	165	2.7	61.11	132	2.16	0.069	0.046
0.5	670	2.1	319.05	510	1.60	0.103	0.084
0.6	546	1.7	321.18	415	1.29	0.151	0.129
0.7	468	1.5	312.00	340	1.09	0.206	0.182
0.8	357	1.1	324.55	300	0.92	0.269	0.253
0.9	350	1.1	318.18	265	0.83	0.340	0.311
1	509	0.8	636.25	452	0.71	0.425	0.428
1.5	536	0.8	670.00	336	0.50	0.945	0.953
2	509	0.8	636.25	268	0.42	1.670	1.720
2.5	351	0.4	877.50	212	0.24	2.638	2.665
**Rotation about y-axis for 45°**							
Current (A)	Total video Frames	Total video duration (s)	Frame rate (s^{-1})	Effective rotating frames	Rotating time (s)	Theoretical maximum torque ($\times10^{-5}$ N•m)	Experimental maximum torque ($\times10^{-5}$ N•m)
0.1	537	9	59.67	480	8.04	0.010	0.011
0.2	248	4.1	60.49	237	3.92	0.037	0.046
0.3	174	2.9	60.00	158	2.63	0.086	0.101
0.4	134	2.2	60.91	122	2.00	0.151	0.175
0.5	111	1.8	61.67	100	1.62	0.224	0.267
0.6	442	1.4	315.71	412	1.30	0.330	0.413
0.7	344	1.1	312.73	344	1.10	0.449	0.581
0.8	455	1.4	325.00	362	1.11	0.586	0.567
0.9	376	1.2	313.33	278	0.89	0.742	0.894
1	615	1	615.00	579	0.94	0.924	0.961
1.5	528	0.8	660.00	385	0.58	2.062	2.067
2	461	0.7	658.57	288	0.44	3.643	3.678
2.5	4504	5.6	804.29	272	0.34	5.754	6.149
**Rotation about z-axis for 45°**							
Current (A)	Total video Frames	Total video duration (s)	Frame rate (s^{-1})	Effective rotating frames	Rotating time (s)	Theoretical maximum torque ($\times10^{-5}$ N•m)	Experimental maximum torque ($\times10^{-5}$ N•m)
0.1	856	14.4	59.44	740	12.45	0.006	0.005
0.2	389	6.5	59.85	373	6.23	0.020	0.020
0.3	236	3.9	60.51	227	3.75	0.046	0.052
0.4	178	3	59.33	169	2.85	0.082	0.091
0.5	742	2.4	309.17	685	2.22	0.121	0.150
0.6	610	2	305.00	561	1.84	0.179	0.217
0.7	450	1.5	300.00	455	1.52	0.243	0.320
0.8	468	1.5	312.00	416	1.33	0.318	0.414
0.9	409	1.3	314.62	376	1.20	0.402	0.515
1	778	1.3	598.46	730	1.22	0.502	0.494
1.5	804	0.9	893.33	723	0.81	1.117	1.122
2	807	0.9	896.67	540	0.60	1.973	2.028
2.5	602	0.6	1003.33	497	0.50	3.116	2.998

Supplementary Table 1 | Experimental data for validation of maximum magnetic torques about three principal axes of the soft magnet. We shot the video of the soft magnet that rotates about three principal axes for 45° to align with the magnetic field and recorded the time of rotation to

estimate the maximum torque. The experiments are conducted under different magnitudes of the applied field by tuning current in the coils. For each video, we calculate its frame rate dividing the total video frames by the total video duration. Then the effective rotation frames are counted. Finally, the rotation time is calculated according to the frame rate and the effective rotation frames. The method of estimating the maximum torque from rotation time is given by the section of "Method" in the main text. The moment of inertia of the capsule robot including the soft magnet about **a**-axis, **b**-axis, **c**-axis are $1{,}255.849\times10^{-9}$ kg·m^2, $4{,}092.301\times10^{-9}$ kg·m^2, $4{,}278.615\times10^{-9}$ kg·m^2, respectively.

Supplementary Notes

Supplementary Note 1: Demagnetizing factors of anisotropic ellipsoids, cuboids and elliptic cylinders.

1. Demagnetization factors of an anisotropic triaxial ellipsoid

To compute demagnetization factors of an anisotropic ellipsoid, we employ the method provided in [1]. Firstly, the following assumption is made:

$$a > b > c > 0 \tag{1}$$

where *a*, *b*, and *c* are the semi-axial lengths of the ellipsoid's three principal axes. The corresponding demagnetization factors are n_a, n_b and n_c. The equations to calculate n_a, n_b and n_c are given below:

$$n_a = \frac{4\pi\cos(\varphi)\cos(\theta)}{\sin^3(\theta)\sin^2(\alpha)}[F(k,\theta) - E(k,\theta)] \tag{2}$$

$$n_b = \frac{4\pi\cos(\varphi)\cos(\theta)}{\sin^3(\theta)\sin^2(\alpha)\cos^2(\alpha)}[E(k,\theta) - cos^2(\alpha)F(k,\theta) - \frac{\sin^2(\alpha)\sin(\theta)\cos(\theta)}{\cos(\varphi)}] \tag{3}$$

$$n_c = \frac{4\pi\cos(\varphi)\cos(\theta)}{\sin^3(\theta)\cos^2(\alpha)}[\frac{\sin(\theta)\cos(\varphi)}{\cos(\theta)} - E(k,\theta)] \tag{4}$$

where

$$\cos(\theta) = \frac{c}{a}, (0 \le \theta \le \frac{\pi}{2}) \tag{5}$$

$$\cos(\varphi) = \frac{b}{a}, (0 \le \varphi \le \frac{\pi}{2}) \tag{6}$$

$$\sin(\alpha) = [\frac{1-(b/a)^2}{1-(c/a)^2}]^{\frac{1}{2}} = \frac{\sin(\varphi)}{\sin(\theta)} = k,, (0 \le \alpha \le \frac{\pi}{2}) \tag{7}$$

$F(k,\theta)$, $E(k,\theta)$ are incomplete elliptic integrals of the first and second kind, k is the modulus and θ is the amplitude of these integrals. And n_a, n_b and n_c follow the rule that:

$$n_a + n_b + n_c = 1 \tag{8}$$

2. Demagnetization factors of an anisotropic cuboid

The formulas for calculating the demagnetization factors of a cuboid are given below [2]:

$$n_c = \frac{b^2-c^2}{2\pi bc}\ln\left(\frac{\sqrt{a^2+b^2+c^2}-a}{\sqrt{a^2+b^2+c^2}+a}\right) + \frac{a^2-c^2}{2\pi ac}\ln\left(\frac{\sqrt{a^2+b^2+c^2}-b}{\sqrt{a^2+b^2+c^2}+b}\right)$$
$$+\frac{b}{2\pi c}\ln\left(\frac{\sqrt{a^2+b^2}+a}{\sqrt{a^2+b^2}-a}\right) + \frac{a}{2\pi c}\ln\left(\frac{\sqrt{a^2+b^2}+b}{\sqrt{a^2+b^2}-b}\right) + \frac{c}{2\pi a}\ln\left(\frac{\sqrt{b^2+c^2}-b}{\sqrt{b^2+c^2}+b}\right)$$
$$+\frac{c}{2\pi b}\ln\left(\frac{\sqrt{a^2+c^2}-a}{\sqrt{a^2+c^2}+a}\right) + \frac{2}{\pi}\arctan\left(\frac{ab}{c\sqrt{a^2+b^2+c^2}}\right) + \frac{a^3+b^3-2c^3}{3\pi abc}$$
$$+\frac{a^2+b^2-2c^2}{3\pi abc}\sqrt{a^2+b^2+c^2} + \frac{c}{\pi ab}\left(\sqrt{a^2+c^2}+\sqrt{b^2+c^2}\right)$$

$$-\frac{(a^2+b^2)^{\frac{3}{2}}+(b^2+c^2)^{\frac{3}{2}}+(c^2+a^2)^{\frac{3}{2}}}{3\pi abc} \quad (9)$$

where $2a$, $2b$, and $2c$ represent the length, width, and height of the cuboid, respectively, and n_c denotes the demagnetization factor along the axis of height. By replacing c with b, b with a, and a with c in Equation (9), the formula for calculating n_b is obtained. Similarly, by replacing c with a, a with b, and b with c in Equation (9), the formula for calculating n_a is obtained. n_a, n_b and n_c also follow Equation (8).

3. Demagnetization factors of an anisotropic elliptic cylinder

Consider an elliptic cylinder with semi-axial length a and b, thickness (height) t. Assume that $a \geq b$ and define the ratio $\beta = \frac{b}{a} \leq 1$. n_a, n_b and n_t are demagnetization factors along three dimensions of the elliptic cylinder. The parameters employed in calculation of demagnetization factors for elliptic cylinders are summarized in Supplementary Table 2.

Symbol	Value	Range	Description
a		$(0,+\infty)$	Major semi-axial length
b		$(0,a]$	Minor semi-axial length
t		$(0,+\infty)$	Thickness (height)
ϵ	$\sqrt{1-(b/a)^2}$	$[0,1)$	Eccentricity
ξ	$t/2b$	$(0,+\infty)$	Secondary aspect ratio
k_ξ	$1/\sqrt{1+\xi^2}$	$(0,1)$	

Supplementary Table 2 | Symbols and parameters characterizing the elliptic cylinder

The formulas for calculation of magnetization factors of elliptical cylinders are given by [3]:

$$n_t = \sum_{n=0}^{\infty} u_n(\xi)\epsilon^{2n} \quad (10)$$

$$n_a = \sum_{n=0}^{\infty} v_n(\xi)\epsilon^{2n} \quad (11)$$

We use the explicit first six terms of $u_n(\xi)$ and $v_n(\xi)$ to calculate a good approximation value of n_t and n_a:

$$u_0(\xi) = 1 + \frac{4}{3\pi\xi}\left\{1 - \frac{1}{k_\xi}[(1-\xi^2)E(k_\xi^2) + \xi^2 K(k_\xi^2)]\right\};$$

$$u_1(\xi) = \frac{1}{3\pi\xi}\left\{1 - \frac{1}{k_\xi}[(1+2\xi^2)E(k_\xi^2) - 2\xi^2 K(k_\xi^2)]\right\};$$

$$u_2(\xi) = \frac{3}{16\pi\xi}\left\{1 - \frac{1}{k_\xi}\left[E(k_\xi^2) - k_\xi^2\xi^2K(k_\xi^2)\right]\right\};$$

$$u_3(\xi) = \frac{5}{192\pi\xi}\{5 - k_\xi[(5 + 4\xi^2)E(k_\xi^2) - 4K(k_\xi^2)]\}; \quad (12)$$

$$u_4(\xi) = \frac{35}{12288\pi\xi}\{35 - k_\xi^3[(5 + 3\xi^2)(7 + 8\xi^2)E(k_\xi^2)$$
$$-\xi^2(25 + 4\xi^2)K(k_\xi^2)]\};$$

$$u_5(\xi) = \frac{21}{81920\pi\xi}\{315 - k_\xi^5[(315 + 875\xi^2 + 742\xi^4 + 196\xi^6)E(k_\xi^2)$$
$$-2\xi^2(105 + 203\xi^2 + 96\xi^4)K(k_\xi^2)]\}$$

$$v_0(\xi) = -\frac{2}{3\pi\xi}\left\{1 - \frac{1}{k_\xi}\left[(1 - \xi^2)E(k_\xi^2) + \xi^2K(k_\xi^2)\right]\right\};$$

$$v_1(\xi) = -\frac{1}{12\pi\xi}\left\{1 - \frac{1}{k_\xi}\left[(1 + 8\xi^2)E(k_\xi^2) - 8\xi^2K(k_\xi^2)\right]\right\};$$

$$v_2(\xi) = -\frac{1}{32\pi\xi}\left\{1 - \frac{1}{k_\xi}\left[E(k_\xi^2) - 5k^2\xi^2K(k_\xi^2)\right]\right\};$$

$$v_3(\xi) = -\frac{5}{1536\pi\xi}\{5 - k_\xi[(5 - 2\xi^2)E(k_\xi^2) - 22\xi^2K(k_\xi^2)]\}; \quad (13)$$

$$v_4(\xi) = -\frac{7}{24576\pi\xi}\{35 - k_\xi^3[(35 - 3\xi^2 - 56\xi^4)E(k_\xi^2)$$
$$-\xi^2(145 + 136\xi^2)K(k_\xi^2)]\};$$

$$v_5(\xi) = -\frac{7}{327680\pi\xi}\{315 - k_\xi^5[(315 + 147\xi^2 - 928 - 848\xi^6)E(k_\xi^2)$$
$$-4\xi^2(315 + 594\xi^2 + 268\xi^4)K(k_\xi^2)]\}.$$

where $K(k_\xi^2)$ and $E(k_\xi^2)$ are complete elliptic integrals of the first and the second kind. Finally, n_b can be calculated by the unity constraint:

$$n_b = 1 - n_a - n_t \quad (14)$$

Supplementary Note 2: Preliminary results on modeling of 3-DoF magnetic force and 6-DoF manipulation of soft-magnet robots

1. Modeling of 3-DoF magnetic force on an anisotropic soft magnet

Magnetic force on a magnetic dipole **m** depends on the spatial gradient of the local magnetic field **B**, which is given below

$$\mathbf{f} = \nabla(\mathbf{B}\cdot\mathbf{m}) = \begin{bmatrix} \frac{\partial \mathbf{B}}{\partial \mathrm{x}} & \frac{\partial \mathbf{B}}{\partial \mathrm{y}} & \frac{\partial \mathbf{B}}{\partial \mathrm{z}} \end{bmatrix}^{\mathrm{T}} \mathbf{m} = \begin{bmatrix} m_x \frac{\partial B_x}{\partial \mathrm{x}} + m_y \frac{\partial B_y}{\partial \mathrm{x}} + m_z \frac{\partial B_z}{\partial \mathrm{x}} \\ m_x \frac{\partial B_x}{\partial \mathrm{y}} + m_y \frac{\partial B_y}{\partial \mathrm{y}} + m_z \frac{\partial B_z}{\partial \mathrm{y}} \\ m_x \frac{\partial B_x}{\partial \mathrm{z}} + m_y \frac{\partial B_y}{\partial \mathrm{z}} + m_z \frac{\partial B_z}{\partial \mathrm{z}} \end{bmatrix} \tag{15}$$

Now replace the magnetic dipole with an anisotropic soft magnet, with **a-**, **b-**, and **c-**axis of the soft magnet aligned with the **x-**, **y-**, and **z-**axis of the world coordinate system (WCS) as shown in Supplementary Fig. 7 (a). The soft magnet's magnetization is determined by Equation (3) - (7) in the paper. Combining these equations with Equation (15), we can obtain magnetic force **f** on the soft magnet in an external magnetic field **B**:

$$\mathbf{f} = \frac{v}{\mu_0}(\mathbf{NB}\cdot\nabla)\mathbf{B} = \frac{v}{\mu_0}\begin{bmatrix} \frac{B_x}{n_a}\frac{\partial B_x}{\partial \mathrm{x}} + \frac{B_y}{n_b}\frac{\partial B_y}{\partial \mathrm{x}} + \frac{B_z}{n_c}\frac{\partial B_z}{\partial \mathrm{x}} \\ \frac{B_x}{n_a}\frac{\partial B_x}{\partial \mathrm{y}} + \frac{B_y}{n_b}\frac{\partial B_y}{\partial \mathrm{y}} + \frac{B_z}{n_c}\frac{\partial B_z}{\partial \mathrm{y}} \\ \frac{B_x}{n_a}\frac{\partial B_x}{\partial \mathrm{z}} + \frac{B_y}{n_b}\frac{\partial B_y}{\partial \mathrm{z}} + \frac{B_z}{n_c}\frac{\partial B_z}{\partial \mathrm{z}} \end{bmatrix} \tag{16}$$

where v is the volume of the soft magnet and μ_0 is the permeability in vacuum. From Equation (16), to apply a desired force to the soft magnet, we need to provide both an appropriate magnetic field and an appropriate magnetic field spatial gradient. Equation (16) also shows that it is feasible to generate magnetic forces on the soft magnet in three directions for 3-DoF translation control.

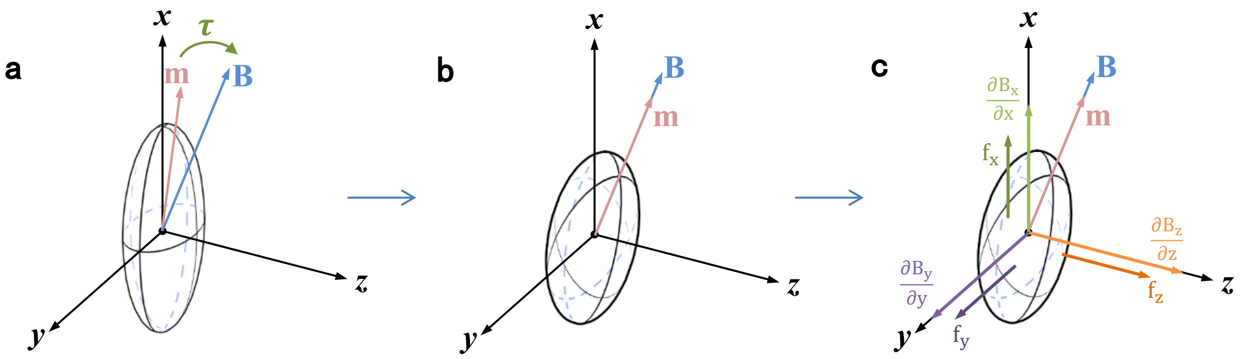

Supplementary Fig. 7 | Magnetic force on soft magnet in a magnetic field with spatial gradient. **a.** Schematic of anisotropic magnetization and magnetic torque of a tri-ellipsoidal soft magnet. The anisotropic magnetization of the soft magnet leads to separation of the direction of magnetization vector **m** from the applied field **B**, resulting in a non-zero magnetic torque **τ**. **b.** Alignment of an ellipsoidal soft magnet's **a**-axis with the direction of magnetic field **B** due to the torque **τ**. **c.** Schematic of 3-DoF magnetic force on the soft magnet subjected to both magnetic field **B** and magnetic field gradient ∇**B**.

The orthogonal Helmholtz coils used in the paper can only generate a uniform magnetic field but lacks the capability to produce magnetic field spatial gradients. Therefore, we need to design a new magnetic field generation setup, i.e., the Helmholtz-Maxwell combination coils. The Helmholtz-Maxwell coils consist of three pairs of Helmholtz coils and three pairs of Maxwell coils, both of which are installed orthogonally. A uniform magnetic field can be generated in any direction by Helmholtz coils that runs the currents in the same direction in pairs of coils, while three magnetic field gradients, namely $\frac{\partial B_x}{\partial x}$, $\frac{\partial B_y}{\partial y}$, and $\frac{\partial B_z}{\partial z}$, can be provided by Maxwell coils that runs the currents in the opposite direction in pairs of coils. In the working space of the setup, the magnitude of $\frac{\partial B_y}{\partial x}$, $\frac{\partial \mathrm{B}_z}{\partial \mathrm{x}}$, $\frac{\partial B_x}{\partial \mathrm{y}}$, $\frac{\partial B_z}{\partial \mathrm{y}}$, $\frac{\partial B_x}{\partial \mathrm{z}}$ and $\frac{\partial B_y}{\partial \mathrm{z}}$ are negligible compared to the magnitude of $\frac{\partial B_x}{\partial x}$, $\frac{\partial B_y}{\partial y}$, and $\frac{\partial B_z}{\partial z}$. Therefore, Equation (1) can be simplified to

$$\mathbf{f} = \left[m_x \frac{\partial B_x}{\partial \mathrm{x}} \quad m_y \frac{\partial B_y}{\partial \mathrm{y}} \quad m_z \frac{\partial B_z}{\partial \mathrm{z}}\right]^{\mathrm{T}} \tag{17}$$

As shown in Supplementary Fig. 7 (a) and (b), when **B** is applied in an arbitrary direction without a magnetic field spatial gradient, the soft magnet experiences a torque that causes its **a**-axis to align with the direction of the magnetic field according to the analysis of orientation stability in the paper. Once aligned, the magnetic moment **m** of the soft magnet under **B** is given by:

$$\mathbf{m} = \frac{v}{\mu_0 n_a}\mathbf{B} \tag{18}$$

Substitute Equation (18) into (17), the following equation of magnetic force is obtained:

$$\mathbf{f} = \frac{v}{\mu_0 n_a}\left[B_x \frac{\partial B_x}{\partial \mathrm{x}} \quad B_y \frac{\partial B_y}{\partial \mathrm{y}} \quad B_z \frac{\partial B_z}{\partial \mathrm{z}}\right]^{\mathrm{T}} \tag{19}$$

By analyzing Equation (19), we can draw the following conclusion: Given that **a**-axis of the soft magnet aligns with the direction of **B**, we can control the magnetic force **f** on the soft magnet by regulating the terms $B_x \frac{\partial B_x}{\partial \mathrm{x}}$, $B_y \frac{\partial B_y}{\partial \mathrm{y}}$ and $B_z \frac{\partial B_z}{\partial \mathrm{z}}$ through the Helmholtz-Maxwell combination coils, as shown in Supplementary Fig. 7 (c).

2. A preliminary control method for 6-DoF manipulation

In the following section, we will introduce a control strategy that we are currently conceptualizing for 6-DoF manipulation of the soft-magnet robot. This 6-DoF control strategy is built upon the open-loop 3-DoF orientation control method proposed in this paper.

Supplementary Fig. 8 (a), (b), and (c) illustrate translation control in three directions. Taking translation control in **x**-axis as an example, a magnetic field **B** is applied in **x**-axis to align the **a**-axis of the soft magnet and magnetic moment **m** with the field by activating the Helmholtz coils along the **x**-axis. Then activate the Maxwell coils along the **x**-axis to generate $\frac{\partial B_x}{\partial x}$ while maintaining **B**. According to Equation (19), the force can be expressed as $\mathbf{f} = \frac{v}{\mu_0 n_a}\left[B_x \frac{\partial B_x}{\partial \mathrm{x}} \quad 0 \quad 0\right]^{\mathrm{T}}$. Therefore, **f**

does not depend on $\frac{\partial B_y}{\partial y}$ and $\frac{\partial B_z}{\partial z}$ and we can control the magnetic force in **x**-axis by tuning only $\frac{\partial B_x}{\partial x}$. Similarly, we can control the magnetic force in **y**-axis and **z**-axis by tuning $\frac{\partial B_y}{\partial y}$ and $\frac{\partial B_z}{\partial z}$, respectively, as depicted in Supplementary Fig. 8 (b) and (c). It is noted that a position sensor is needed to provide 3-DoF position of the soft-magnet robot for feedback (closed-loop) translation control.

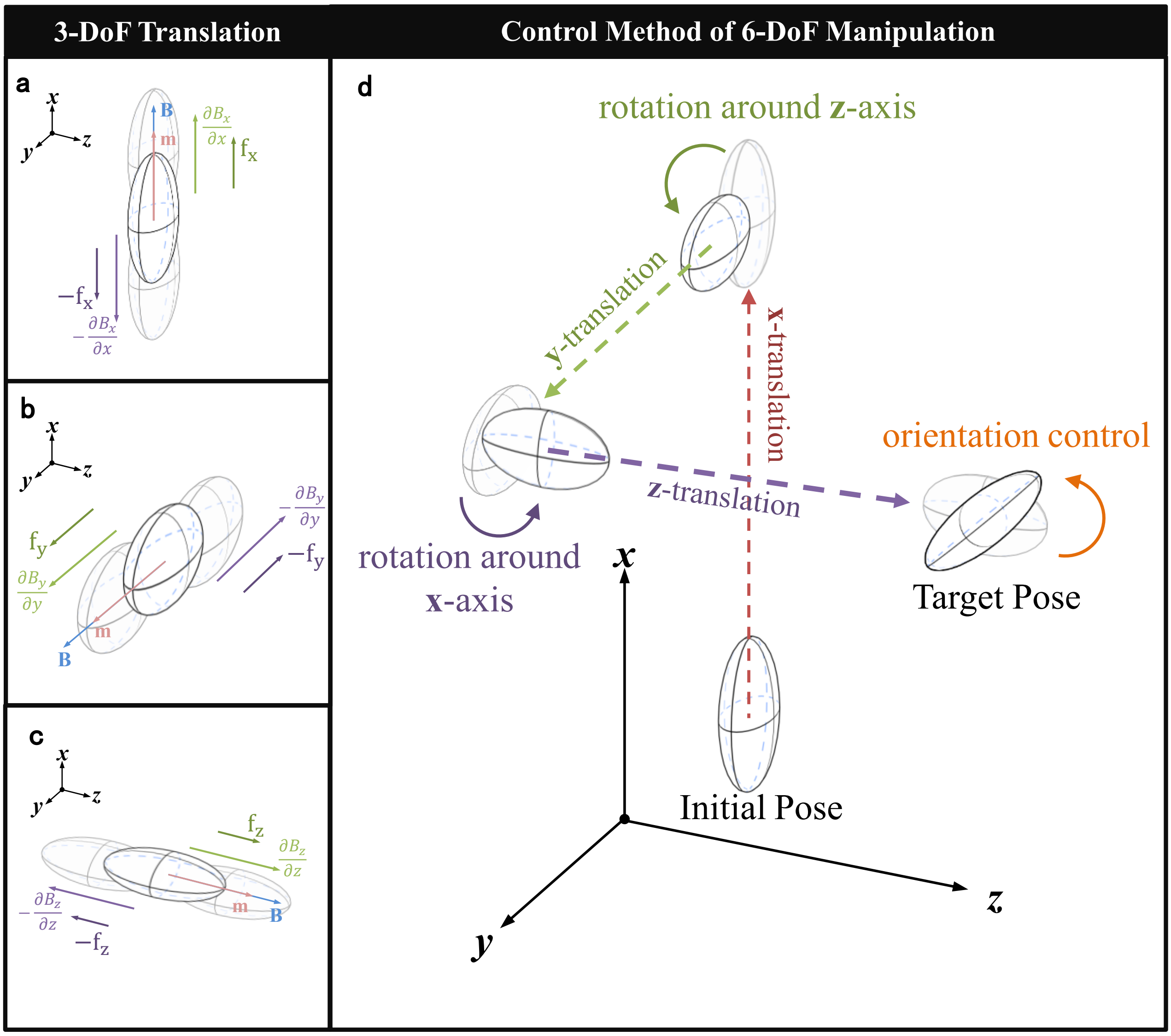

Supplementary Fig. 8 | Translation control and 6-DoF manipulation of an anisotropic soft magnet. a. Translation control in **x**-axis. **b.** Translation control in **y**-axis. **c.** Translation control in **z**-axis. **d.** 6-DoF manipulation of the soft magnet with decomposed translation and orientation control.

6-DoF manipulation can be decomposed into 3-DoF translation and 3-DoF rotation. First, the soft-magnet robot is translated in three directions one at a time. During each translation, magnetic field **B** keeps **a**-axis with the translation direction while $\nabla\mathbf{B}$ provides the force for translation. When shifting among the three translation directions, we need to rotate the soft magnet to align its **a**-axis with the translation direction. For instance, as shown in Supplementary Fig. 8 (d), when the soft-

magnet robot is switched from **x**-axis translation to **y**-axis translation, it needs to rotate 90° about **z**-axis using the proposed open-loop orientation control method to align the longest **a**-axis with the heading direction. Once the soft-magnet robot reaches the target position through translation, we can utilize the orientation control method proposed in the paper to manipulate its orientation. The whole process of 6-DoF manipulation is illustrated in Supplementary Fig. 8 (d).

Supplementary Note 3: The limits of frequency of alternating magnetic control

1. Lower limits of frequency of alternating magnetic control

The frequency of the alternating magnetic control is 10 Hz in the experiments shown in Fig. 6, which is sufficient to rotate the soft-magnet robot smoothly. However, the frequency of magnetic control cannot be too low for two reasons:

(1) Since the proposed open-loop control strategy rotates the soft-magnet robot in two alternating stages, i.e., controlling the direction of the longest axis and controlling the rotation about the longest axis, low frequency of the alternating magnetic control results in non-smooth orientation manipulation, which is not desired for continuous robot control. Supplementary Movie 9 (Section 2) shows the orientation manipulation at 2 Hz, where the soft-magnet robot is rotated in a non-smooth manner.

(2) It is shown that the longest axis of the soft magnet always tends to align with the applied magnetic field (Fig. 4). Lower frequency of magnetic control allows for a longer period of rotation about the longest axis, during which potential disturbances might deflect the longest axis so an undesired magnetic torque is generated to drive the longest axis towards the direction of $\mathbf{B}_i^r$, which is not desired (the longest axis should be pointed to the direction of $\mathbf{B}_i^a$) and might cause instability of orientation manipulation. Although this issue also exists in the case with high frequency of magnetic control, the potential wrong deflection of the longest axis towards $\mathbf{B}_i^r$ lasts for a shorter time and it can be quickly corrected by $\mathbf{B}_i^a$ in the high-frequency case. Supplementary Movie 9 (Section 1) shows the orientation manipulation at 0.5 Hz, where the soft-magnet robot continues to rotate and fails to stabilize at the target orientation.

2. Upper limit of frequency for alternating open-loop control strategy

Theoretically, there is no upper limit of the frequency of magnetic control. However, the inductance effect of the Helmholtz coils cannot be neglected at high frequencies. At high frequencies, although the voltage input to the coils is a square wave, the current and the resulting magnetic field become approximately constant with a magnitude that is determined by the duty cycle. This is exactly the same as the principle of the pulse width modulation (PWM). Therefore, the alternating application of $\mathbf{B}_i^r$ and $\mathbf{B}_i^a$ become a constant application of $(1-a)\mathbf{B}_i^r$ and $a\mathbf{B}_i^a$, where a is the duty cycle of $\mathbf{B}_i^a$ as shown in Supplementary Fig. 9 (a). The final effect of the magnetic control at high frequencies is

equivalent to applying a constant magnetic field $(1-a)\mathbf{B}_i^r + a\mathbf{B}_i^a$, which degenerates to controlling a soft magnet with a single magnetic field (Supplementary Fig. 9 (b)). Since it is shown in the paper that a single magnetic field cannot fully control the 3-DoF orientation of the soft magnet, there indeed exists an upper limit of frequency beyond which the proposed control strategy fails. Supplementary Movie 10 and Supplementary Fig. 9 (c) shows the orientation manipulation at 10 kHz, where the soft magnet aligns its longest axis with the direction of the superimposed magnetic field and fails to reach the target orientation.

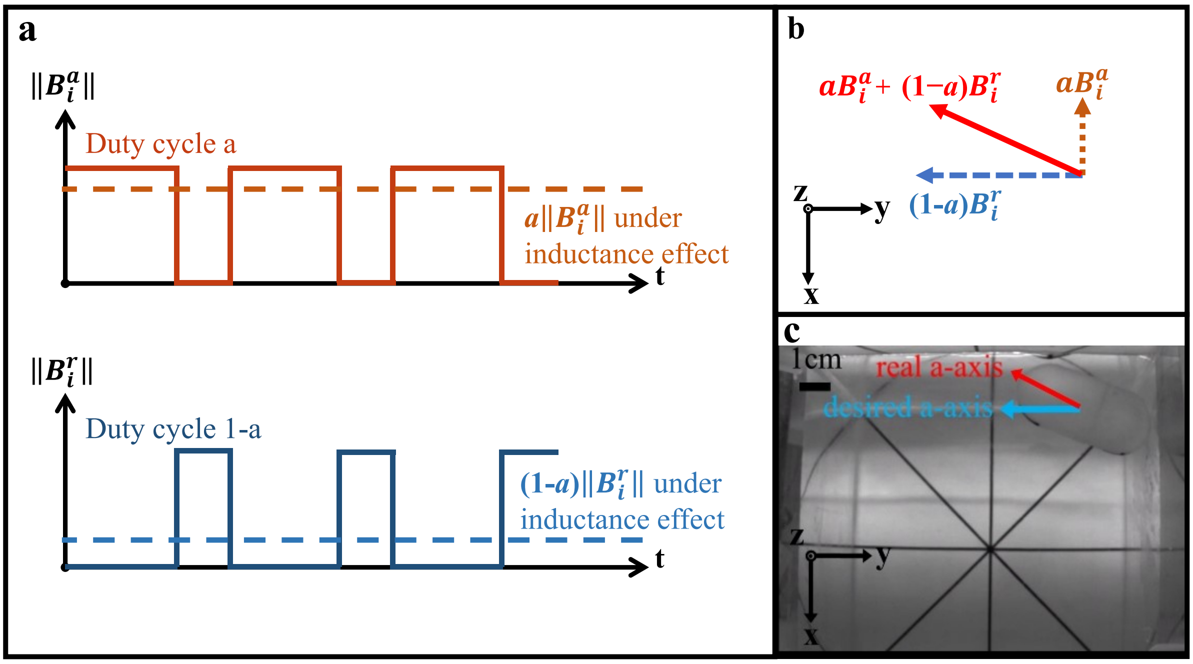

Supplementary Fig. 9 | Upper limit of frequency of alternating magnetic control. a. High-frequency switching of $\mathbf{B}_i^r$ and $\mathbf{B}_i^a$ results in equivalent constant magnetic fields due to the induction effect. **b.** Equivalent superimposed magnetic field $(1-a)\mathbf{B}_i^r + a\mathbf{B}_i^a$ due to the induction effect at high frequencies. **c.** Rotation about **z**-axis at the upper limit of frequency of magnetic control (10 kHz). The capsule robot aligns its a-axis with the direction of the superimposed magnetic field $(1-a)\mathbf{B}_i^r + a\mathbf{B}_i^a$ rather than the desired direction of $\mathbf{B}_i^a$.

Supplementary References

[1] Osborn, J. A. Demagnetizing factors of the general ellipsoid. *Physical review*, *67*(11-12), 351. (1945).

[2] Aharoni, A. Demagnetizing factors for rectangular ferromagnetic prisms. *Journal of applied physics*, *83*(6), 3432-3434. (1998).

[3] Beleggia, M., De Graef, M., Millev, Y. T., Goode, D. A., & Rowlands, G. Demagnetization factors for elliptic cylinders. *Journal of Physics D: Applied Physics*, *38*(18), 3333. (2005).
