# Supplementary material for: Three-degrees-of-freedom orientation manipulation of small untethered robots with a single anisotropic soft magnet
Source: Nat Commun. 2023 Nov 18;14:7491. doi: 10.1038/s41467-023-42783-5 (PMC10657469; doi:10.1038/s41467-023-42783-5)
Supplement: Supplementary file 3 — Description of Additional Supplementary Files [file 41467_2023_42783_MOESM3_ESM.pdf]

Description of Additional Supplementary Files

File name: Supplementary Movie 1.
Description: Orientation stability with a-axis aligned with the applied field.

File name: Supplementary Movie 2.
Description: Orientation stability with b-axis aligned with the applied field.

File name: Supplementary Movie 3.
Description: Orientation stability with c-axis aligned with the applied field.

File name: Supplementary Movie 4.
Description: Orientation control about a-axis of the soft magnet.

File name: Supplementary Movie 5.
Description: Orientation control about b-axis of the soft magnet.

File name: Supplementary Movie 6.
Description: Orientation control about c-axis of the soft magnet.

File name: Supplementary Movie 7.
Description: 3-DoF orientation manipulation over an arbitrary orientation path.

File name: Supplementary Movie 8.
Description: 3-DoF orientation manipulation in the environmental flow field.

File name: Supplementary Movie 9.
Description: Lower limit of frequency of magnetic control for orientation control.

File name: Supplementary Movie 10.
Description: Upper limit of frequency of magnetic control for orientation control.
